# AGE EFFECTS ON WHITE MATTER TOPOLOGY IN OLDER ADULTS AT HIGH RISK OF ALZHEIMER’S DISEASE

**DOI:** 10.1093/geroni/igad104.2230

**Published:** 2023-12-21

**Authors:** Meishan Ai, Emma Tinney, Charles Hillman, Nathan Spreng, Arthur Kramer, Maiya Geddes, PREVENT-AD Research Group

**Affiliations:** Northeastern University, Boston, Massachusetts, United States; Northeastern University, Boston, Massachusetts, United States; Northeastern University, Boston, Massachusetts, United States; McGill University, Montreal, Quebec, Canada; Northeastern University, Boston, Massachusetts, United States; McGill University, Montreal, Quebec, Canada; McGill University, Montreal, Quebec, Canada

## Abstract

White matter integrity shows age-related declines in later life. Studies have found altered topological changes of white matter networks in healthy aging populations. We investigated cross-sectional association between white matter network topology, age, education, and cognition in older adults at high-risk for Alzheimer’s disease (AD). A total of 153 cognitively healthy participants (age=67.78±5.03, 111 female, 54 APOE-4 carriers) who have at least one close family member diagnosed with AD from the Pre-symptomatic Evaluation of Experimental or Novel Treatments for Alzheimer’s Disease (PREVENT-AD) cohort were included in the study. Whole brain diffusion images were obtained and submitted to preprocessing, fiber tracking and connectome creation in MRtrix. Test scores from the Trail Making task, Stroop task, and Rey Auditory Verbal Learning Test (RAVLT) were included as cognitive function measures. We found that older age was significantly associated with greater modularity (r2=0.48, p corrected< 0.001), lower density (r2=0.32, p corrected< 0.001), greater betweenness centrality (r2=0.39, p corrected=0.003), and greater clustering coefficient (r2=0.05, p corrected=0.042). Older age was also significantly associated with longer Trail Making time series B (r2=0.11, p corrected=0.012) and fewer recalled words (r2=0.18, p corrected=0.007). We further found that participants with higher educational attainment showed less age-related changes in modularity (t=2.42, p=0.017) and density (t=2.40, p=0.018). However, these graph theory measures were not associated with cognitive performance. In conclusion, age related to whole-brain white matter network topology in high-risk healthy older adults, and education may attenuate this relationship. The current study deepens our understanding of white matter changes in high-risk aging.

